# Evaluation of curricular relevance and actual integration of sex/gender and cultural competencies by final year medical students: effects of student diversity subgroups and curriculum

**DOI:** 10.3205/zma001312

**Published:** 2020-03-16

**Authors:** Sabine Ludwig, Susanne Dettmer, Wiebke Wurl, Ute Seeland, Asia Maaz, Harm Peters

**Affiliations:** 1 Charité - Universitätsmedizin Berlin, Berlin, Germany; 2 Charité - Universitätsmedizin Berlin, Institute of Medical Sociology and Rehabilitation Science, Berlin, Germany; 3 Charité - Universitätsmedizin Berlin, Institute of Gender in Medicine, Berlin, Germany; 4 Charité - Universitätsmedizin Berlin, Dean's Office of Student Affairs, Dieter Scheffner Center for Medical Education and Educational Research, Berlin, Germany

**Keywords:** gender, diversity, culture, medical education, student evaluation, medical competencies

## Abstract

**Background: **Diversity issues play a key role in medical practice and have recently been more explicitly integrated into undergraduate medical curricula in Europe and worldwide. However, research on students´ perspectives on the relevance and curricular integration of diversity issues, such as sex/gender and culture-sensitive competencies, is still limited.

**Methods: **The Charité Berlin (Germany) ran in parallel a traditional and a competency-based medical program. Diversity perspectives, especially sex and gender aspects, were systematically integrated into the new curriculum. In 2016, an online questionnaire was sent to all medical students in their final clerkship year of both programs. Students provided diversity-related information (sex/gender, age, number of children, migration background or disability) and rated the relevance of sex/gender and culture-sensitive competencies and the integration into their study program. They also rated their preparedness for the final year clerkships and for working as a physician.

**Results: **The included 184 students considered sex/gender and culture-sensitive competencies to be very relevant or relevant (62%; 73%). The ratings of the relevance are independent of the curriculum and significantly higher in female students. Regarding curricular integration, 69% of the students of the traditional curriculum evaluated the degree of implementation as minor, whereas 83% students of the new curriculum rated the degree of implementation as extensive. Degrees of preparedness for the workplace were significantly higher in students from the new curriculum, with no significant effects by sex/gender. Age group, having a child, migration background or a disability had separate effects on the students’ ratings.

**Conclusions:** Medical students in their final clerkship year rated sex/gender and culture-sensitive competencies as relevant; this was independent from their study program. Their ratings provide complementary evidence that our systematic approach to implementation resulted in a successful curricular integration.

## Zusammenfassung

**Hintergrund: **Diversitätsaspekte spielen in der medizinischen Versorgung eine wichtige Rolle und werden vermehrt in medizinische Curricula in Europa und weltweit integriert. Forschung zur Sichtweise der Studierenden hinsichtlich der Relevanz und der Integration von Diversitätsaspekten wie geschlechter- und kultursensible Kompetenzen ist jedoch noch begrenzt.

**Methodik: **An der Charité – Universitätsmedizin Berlin wurden parallel ein Regel- und ein Modellstudiengang angeboten. Diversitätsperspektiven, insbesondere Geschlechteraspekte, wurden systematisch in den Modellstudiengang integriert. Im Jahr 2016 wurde ein Online- Fragebogen an alle Medizinstudierenden im letzten Studienjahr beider Studiengänge verschickt. Die Studierenden machten diversitätsbezogene Angaben (Geschlecht, Alter, Anzahl der Kinder, Migrationshintergrund, körperliche Beeinträchtigungen / Behinderung) und beurteilten die Relevanz von geschlechter- und kultursensiblen Kompetenzen und deren Integration in ihren Studiengang. Sie beurteilten auch ihren Grad der Vorbereitung auf das Praktische Jahr und auf ihre ärztliche Tätigkeit.

**Ergebnisse: **Die befragten 184 Studierenden betrachteten geschlechter- und kultursensible Kompetenzen als sehr relevant oder relevant (62%; 73%). Die Beurteilung der Relevanz war dabei unabhängig vom Studiengang und signifikant höher bei Studentinnen. Von den Studierenden des Regelstudiengangs bewerteten 69% das Ausmaß der Integration als gering, wobei 83% der Studierenden des Modellstudiengangs das Ausmaß der Integration als groß beurteilten. Die Studierenden des Modellstudiengangs fühlten sich signifikant besser auf die ärztliche Tätigkeit vorbereitet. Dabei waren keine signifikanten Geschlechterunterschiede zu verzeichnen. Alter, Anzahl der Kinder, Migrationshintergrund oder körperliche Beeinträchtigungen / Behinderung hatten separate Effekte auf die Beurteilungen der Studierenden.

**Schlussfolgerung:** Medizinstudierende im letzten Studienjahr beurteilten geschlechtersensible und kulturelle Kompetenzen als relevant, dies war unabhängig vom Studiengang. Diese Beurteilungen liefern zusätzliche Evidenz, dass unsere systematische Vorgehensweise der curricularen Integration erfolgreich war.

## 1. Introduction

Undergraduate medical education is currently in a transition from traditional, discipline-based to integrated, competency-based programs [1], [2], [3]. This is paralleled with the inclusion of diversity-related content and competencies, especially aspects of sex and gender medicine, as well as cultural background [4], [5], [6]. In medical schools in America [7], [8] and Canada [9], [10], [https://cihr-irsc.gc.ca/e/8673.html], there have been several efforts to develop sex and gender-sensitive curricula [11], [12], [https://www.ttuhsc.edu/medicine/sex-gender-specific-health/]. In Europe, sex and gender aspects have been integrated into medical programs e.g. in Austria [13], Sweden [14] and the Netherlands [15]. Verdonk et al. have developed a gender awareness scale [16] and Dogra et al. have published twelve tips for cultural diversity education [17]. Several medical schools in America have made efforts to integrate cultural competencies [18], [19], [20], [21], [22], [23]. In 2014, the Lancet Commission on Culture and Health emphasized the importance of integrating cultural competencies into medical education [24]. The Committee on Cultural Competence and Global Health of the German Association for Medical Education has published a position paper on the integration of cultural competencies into health professions education [25]. So far, little is known about what medical students actually think about the integration of diversity-related competencies into their training program. The purpose of this study is to analyse how medical students evaluate the relevance of diversity-related competencies, such as sex/gender and cultural competencies, and how well this is implemented into their study program. 

In this article we use the following definitions for sex, gender and cultural competency: Sex is defined as the biological factors (e.g. genes, hormones) whereas gender includes the sociocultural factors (e.g. life style, behaviour). In the medical field, sex and gender cannot be properly separated: sex influences health by modifying behaviour and lifestyle and gender-behaviour can modify biological factors and thereby health [26].

Cultural competency is defined as “a collection of ways of thinking and acting acquired in a lifetime and acknowledging that cultural identity depends on context” [25]. 

Diversity issues play a key role in many, if not almost all aspects of our current medical practice [27]. In order to improve the quality of medical care for women and men, it is important that diversity perspectives, such as sex and gender, cultural background, religion, age, disabilities and sexual orientation [28] are integrated into medical curricula as well as into other health professions curricula [13]. Future health professionals should have adequate knowledge on relevant diversity aspects of diseases, for instance sex and gender differences, in the prevention, development, diagnosis, manifestation and treatment of diseases [26], [28]. Along this line, they also need to know gender differences in health behaviour and sex and gender-associated risk factors for specific diseases [8]. Furthermore, health professionals need to be trained in diversity-sensitive communication, in sex and gender differences in public health and also be able to reflect their own gender role [29], [30].

In 2010, at our institution, the Charité - Universitätsmedizin Berlin (Charité), the traditional, discipline-based undergraduate medical program was replaced by a fully integrated, competency-based program (Modular Curriculum of Medicine) [2], [3]. With the introduction of the new program, diversity issues focusing on sex and gender aspects were systematically integrated as teaching content and learning objectives. There are courses on gender medicine, e.g. on sex/gender differences in pharmacology as well as courses with integrated sex and gender learning objectives: “The students shall be able to explain sex and gender differences for type 2 diabetes mellitus, lung cancer and cardiovascular diseases” and “The students shall be able to conduct a gender-sensitive anamnesis, gender-sensitive diagnosis and therapy (9. Semester, Module 35 “Gender-specific Diseases”). Cultural aspects are also integrated, e.g. in courses on the influence of culture and gender on nutritional behaviour (Module 12 “Nutrition, Digestion, Metabolism”), on the medical care for migrants as well as on intercultural aspects in doctor patient communication (2. Semester, Module 6 “Health and Society”) [31]. 

This process was facilitated by a specifically appointed diversity and gender change agent as well as a systematic faculty-transparent ten-step approach for the integration [31]. From a structural point of view and based on the current evaluation standards, this integration can be considered successful [15]. With this approach it could be achieved that 5% of the learning objectives, 21% of the lectures, 12% of the seminars and 8% of the practical courses explicitly encompass diversity issues, especially sex and gender aspects [31]. Beyond the indications for a successful structural integration, we currently do not know what the students – as one main target group – think of this intervention, the relevance of diversity-related competencies for their training and future work as a physician, and to what extent they consider that this is covered via the change of the undergraduate medical program. 

This study aims at addressing the following questions:

1. How do female and male medical students in their final study year evaluate the relevance of diversity-related sex/gender and cultural competencies for their training?
2. To what extent are gender and cultural competencies integrated into the new undergraduate medical program when compared to the previous traditional curriculum?
3. How do female and male medical students rate their preparedness for the final year clerkships and their future work as a physician in the traditional and the new, competency-based curriculum?
4. Are there any differences in the evaluation of the relevance, curricular integration and preparedness between students of further diversity-related student subgroups, such as age group, having a child, migration background or disability?

## 2. Methods

### Setting 

The survey was conducted at the Charité, Berlin, Germany from June to August 2016 using an online questionnaire. The Charité data protection office and ethics board approved the study (No. 8-16; Data Protection and Ethics Board Charité, Campus Mitte). 

The undergraduate medical program encompasses a total of six years. Approximately 600 new students are enrolled each year. In 2010, the former traditional, discipline-based undergraduate medical program was semester-by-semester replaced by a fully integrated, competency-based program for the newly enrolled students [2], [3]. In both programs, the last study year consists of a similar series of clinical clerkships. 

#### Questionnaire development 

A half-standardized online questionnaire was developed in an interdisciplinary iterative process and enclosed faculty members from the quality assurance section of the Office of the Dean of Student Affairs, the Institute of Medical Sociology and Rehabilitation Science, the Dieter Scheffner Center for Medical Education and Educational Research, gender and diversity experts, and medical students. The aim of the survey was to evaluate the quality of teaching and learning, the study environment and curricular structures. Several items of the cooperation project graduate surveys [32] were integrated. With regard to this article, students were asked to provide basic diversity-related demographic information (sex/gender, age, number of children, migration background, disabilities). For the relevance and degree of integration of medical competencies, items from the questionnaires of the study “Career and Life Planning in Medicine” [33] were used: “Please evaluate how relevant the following medical competencies are for you personally” and “please evaluate the degree of curricular integration of the following medical competencies into the medical study program at Charité”. Items on sex/gender and culture-sensitive competencies were added to this instrument. Students were also surveyed on their preparedness for their final year clerkships and their work as a physician. For the items presented in this manuscript (relevance, degree of integration or preparedness), we used 5-point Likert scales for closed questions format (relevance: very relevant, relevant, undecided, not relevant, not relevant at all; integration: very extensive, extensive, undecided, small, very small; preparedness: fully agree, agree, undecided, disagree, completely disagree).

An electronic version of the questionnaire was programmed in the evaluation system EvaSys (Evaluationssysteme GmbH, Lüneburg, Germany). In December 2015 a pre-test was conducted and further modifications based on the feedback from the pre-test were integrated. The modifications did not concern the items studied in this manuscript.

#### Questionnaire administration 

In summer 2016, the questionnaire was sent by email to all medical students (n=835) in their final clerkship year (492 female and 343 male students). This student population was purposively chosen as they have a good overview on the teaching and learning in their study program and already have a reasonably good sense of what is required for their future work as a physician. The questionnaire was sent to 612 students of the traditional curriculum of medicine and to 223 students of the New Modular Curriculum of Medicine at the Charité. Information on the survey was communicated via the Charité students’ association, social media and posters on the campus. The students received several reminders to participate in the survey. The survey period was extended in order to increase the response rate. 

#### Statistics

Statistical data analyses were conducted using SPSS® Statistics 25.0 (IBM, Böblingen, Germany). Descriptive statistics involve the percentage of students’ participation and ratings of items. Where adequate, the mean, median and standard deviations were added. Significance of differences was calculated using the Pearson´s chi-squared test, the Fisher’s exact test, the Kruskal-Wallis test and/or the Mann-Whitney U test. A p-value of <0.05 was considered statistically significant.

## 3. Results

### Study participants 

A total of 184 final year students responded to the survey (22%). Of these, 182 indicated their sex/gender: 114 are female (62%), and 67 male students (37%). This corresponds to the female/male ratio of the whole student population of the undergraduate medical programs at the Charité. One student chose the option “others” for their sex/gender (n=1; 1%). Of the whole study population, 120 (65%) of them study in the traditional curriculum and 64 (35%) in the new curriculum. Regarding age distribution of the students who responded to the survey, 5% of the students in the traditional curriculum are between 20 and 25 years old, 81% between 26 and 30 years, 12% between 31 and 40 years and 2% are over 40 years old (see table 1 (Tab. 1)). In comparison, 39% of the students of the Modular Curriculum of Medicine are between 20 and 25 years old, 52% between 26 and 30 years, 6% between 31 und 40 years and 2% over 40 years old. Further participant characteristics are: 10% (n=18) have a child, 2% (n=3) have further family caring tasks, 20,8% (n=38) a migration background, and 35% (n=64) a disability, i.e. 14% (n=26) psychological disorders (psychosis, depression, eating disorders, addiction), 9% (n=17) chronic somatic disorders, 4% (n=7) mobility impairment, 3% (n=5) visual impairment and 1% (n=2) hearing impairment.

#### Competencies

The following section reports the results of the ratings of female and male students of both curricula of the relevance and extent of integration of sex/gender-sensitive medical competencies and culture-sensitive medical competencies in comparison to general medical competencies of a physician.

#### A. Sex/Gender-sensitive medical competencies

##### Ratings of overall relevance

**Figure 1:**
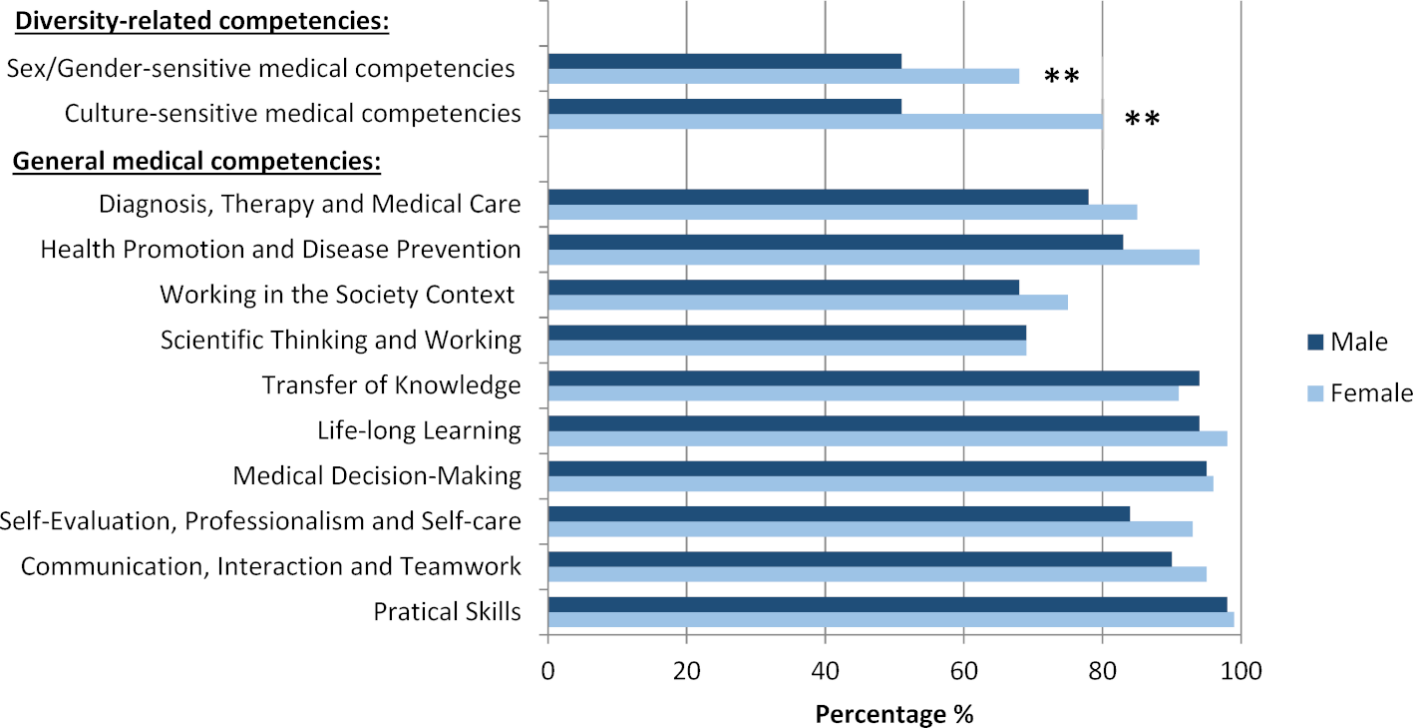
Ratings of the relevance of medical competencies by female and male students of both curricula. The bars indicate the proportion of "very relevant" and "relevant" ratings by the students (male: n=67; female: n=114). The upper part shows diversity-related medical competencies, the lower part general medical competencies. ** indicates p<0.01 male vs. female students.

Overall, 62% (median 2, mean 2,3±0,977 [SD]) of the final year medical students rate sex/gender-sensitive medical competencies “very relevant” or “relevant”. Significantly more female than male students (68% vs. 51%, p<0.01) consider sex/gender-sensitive medical competencies to be “very relevant” or “relevant”. In turn, only 8% of the female students and 16% of the male students do not consider them to be relevant (see figure 1 (Fig. 1)). In addition to sex/gender, there are no significant differences in the ratings of other diversity-related student subgroups such as different age groups, having or not having a migration background, or disabilities. Students with one or more children consider sex/gender-sensitive competencies as more relevant than students without children; however, those differences are not significant (p=0.562). 

##### Ratings of relevance between study programs

Students of both the traditional and the new curriculum generally consider sex/gender-sensitive medical competencies to be highly relevant (63% vs. 62% “very relevant” or “relevant”; traditional curriculum: median 2, mean 2,25±0,97 [SD] ; new curriculum : median 2, mean 2,42±0,98 [SD]). No significant differences between the study programs were noted (p=0.377).

##### Ratings of curricular integration

**Figure 2:**
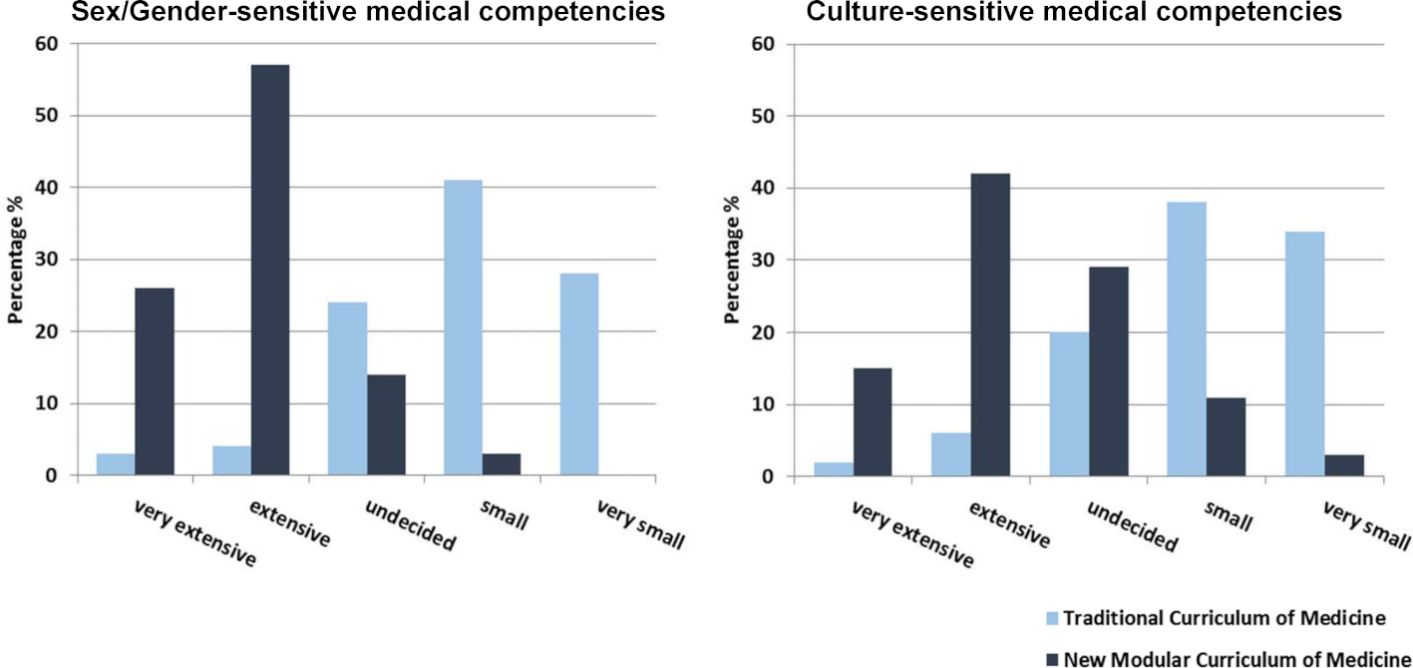
Degrees of curricular integration of sex/gender-sensitive competencies (to the left) and culture-sensitive medical competencies (to the right) present in the traditional and new curriculum. Medical students in the final clerkship year (n=180) rated on a 5-point Likert scale.

Figure 2 (Fig. 2) shows the ratings of curricular integration of sex/gender-sensitive medical competencies of students of the traditional and the new curriculum: Curricular integration was considered to be “very extensive” or “extensive” by only 7% (median 4, mean 3,88±0,96 [SD]) of the students of the traditional curriculum. This is in contrast to the significantly higher ratings of 83% (median 2, mean 1,95±0,72 [SD]) of the students of the New Modular Curriculum of Medicine (p<0.001). In turn, 69% of the students of the traditional curriculum and only 3% of the New Modular Curriculum of Medicine consider the integration to be “very small” or “small”. There are no significant differences apparent in the ratings by female and male students, students of different age groups, having or not having one or more children, migration background or disability on curricular integration.

#### B. Culture-sensitive medical competencies

##### Ratings of overall relevance

Out of the final year medical students participating, 73% (median 2, mean 2,1±0,95 [SD]) rate culture-sensitive medical competencies as “very relevant” or “relevant”. Similar to sex/gender-sensitive medical competencies, significantly more female than male students rate culture-sensitive medical competencies to be “very relevant” or “relevant” (80% vs. 51%, p<0,01) (see figure 1 (Fig. 1)). This corresponds to only 3% of the female students and 19% of the male students who consider culture-sensitive competencies as “not very relevant” or “not relevant at all”. Overall, there are no significant differences in the ratings of students of different age groups, having or not having one or more children, migration background or disability. Students who rate the relevance of sex/gender-sensitive competencies low also rate the relevance of culture-sensitive competencies low, and students who rate the relevance of sex/gender-sensitive competencies high also rate the relevance of culture-sensitive competencies high.

##### Ratings of relevance between study programs

The relevance of culture-sensitive competencies was rated equally high by students from the traditional and the new curriculum (71%, median 2, mean 2,1±0,97 [SD] vs. 76%, median 2, mean 2,11±0,92 [SD]), p=0.163). 

##### Ratings of curricular integration

Figure 2 (Fig. 2) shows the ratings of the extent of curricular integration of culture-sensitive medical competencies of students of the traditional and the new curriculum. Compared to students from the traditional curriculum (8%, median 4, mean 3,97±0,97 [SD]), significantly more students of the New Modular Curriculum of Medicine consider the extent of curricular integration of culture-sensitive medical competencies to be “extensive” or “very extensive” (57%, median 2, mean 2,47±0,99 [SD]; p<0.001). This corresponds to 72% of the students of the traditional curriculum and 14% of the New Modular Curriculum of Medicine who rate the extent of curricular integration as “small” or “very small”. There are no significant differences in the ratings by female and male students on curricular integration, nor in the ratings of students of different age groups, having or not having one or more children, a migration background or a disability.

#### C. Preparedness for the clinical work place

##### Comparison by curriculum

**Figure 3:**
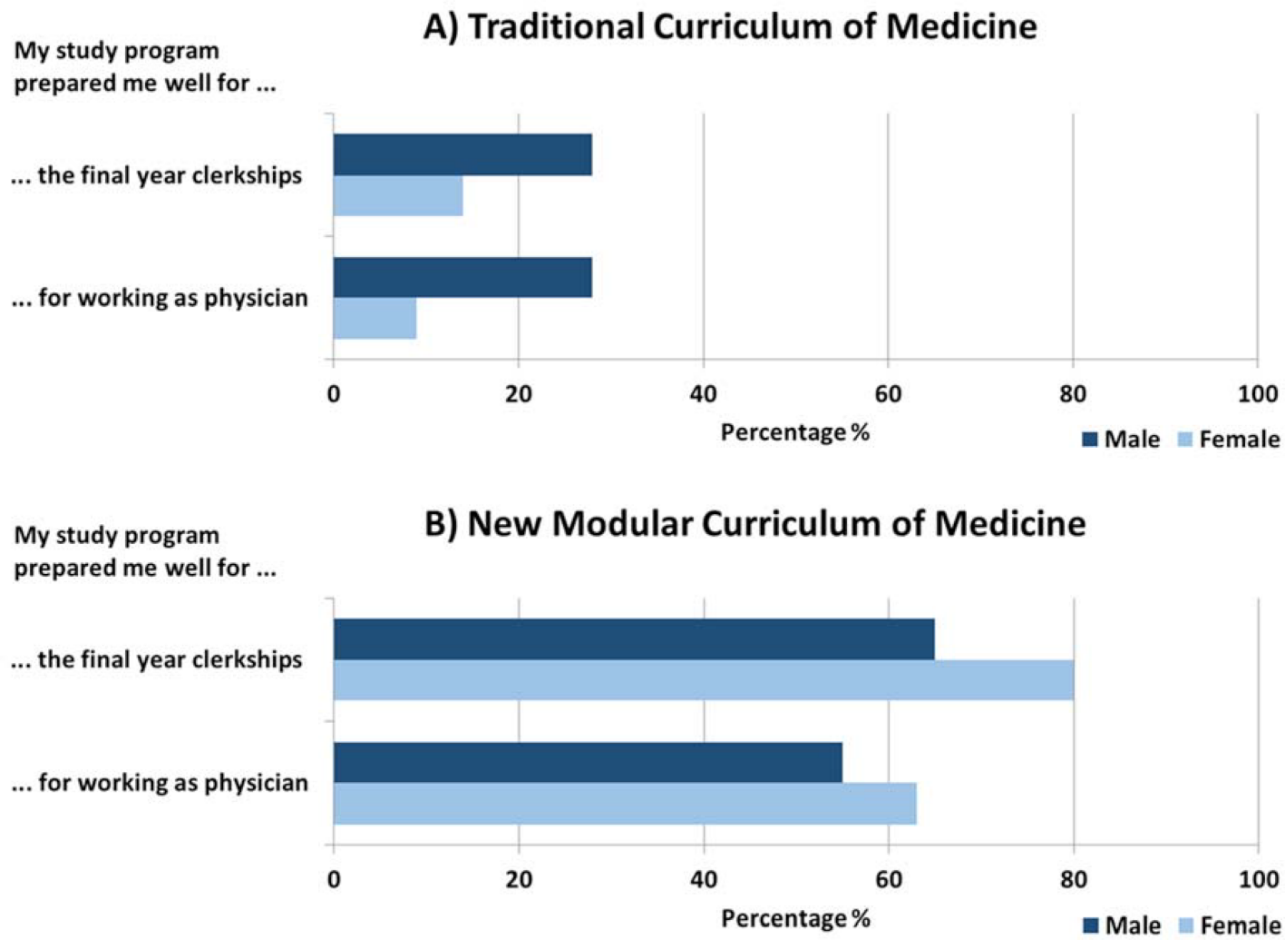
Degrees of preparedness by sex/gender for final year clerkships and working as a physician in the traditional curriculum (A) and in the new curriculum (B). Medical students in the final clerkship year (male: n=67; female: n=114) rated on a 5-point Likert scale.

As depicted in figure 3 (Fig. 3), students from the traditional curriculum rated low their preparedness for the clinical work place, while students from the new curriculum rated their degree of preparedness for the final clerkship year (19%, median 4, mean 3,53±1,1 [SD] vs. 75%, median 2, mean 2,1±0,89 [SD], p<0.001) and their work as physician (16%, median 4, mean 3,47±1,13 [SD]) vs. 60%, median 2, mean 2,46±0,96 [SD]; p<0.001) significantly higher. 

##### Comparison by diversity-related student subgroups

There were some trends, but no significant differences in the ratings of female and male students (see figure 3 (Fig. 3)). In addition, there were no significant differences in the ratings of students of different age groups, having or not having one or more children, a migration background, or a disability.

## 4. Discussion

The implementation of diversity competencies plays an increasing role in the way health care education is conveyed [34], [35]. The recent focus of this implementation has been on structural aspects [36], for instance on questions like “what topics and contents should be included?” “What teaching, learning and assessment formats are appropriate?” “How should they be integrated into already existing or new programs?” “What are the potential quality criteria for a successful integration?” [15], [31]. The present study adds additional and complementary evidence to this development by bringing in the personal perspective of medical students in their final study year on the relevance of diversity-related competencies for their future work as a physician and how well their study program is conveying the necessary learning opportunities for this.

Our study shows that final year medical students to a large degree indicate that sex/gender-sensitive and culture-sensitive medical competencies are indeed considered to be truly relevant for their work as a physician. This is independent of whether or not they were educated and trained in a traditional, discipline-based or an integrated, competency-based program. However, sex/gender-sensitive and culture-sensitive medical competencies are considered somewhat to be less relevant than the general medical competencies for a physician. Overall, this can be seen as a strong and additional argument for the need to implement these competencies into undergraduate medical education in general. This is in our opinion an important message especially as progress in this field is still slow. For instance, a recent study in Germany has shown that only one out of 36 medical faculties had achieved a good integration of sex and gender competencies into their undergraduate medical programs [37].

Of interest is the finding of this study that female students rate the relevance of sex/gender-sensitive and culture-sensitive medical competencies significantly higher than the male students do. This is in line with findings that patient satisfaction is often higher among female physicians [38] and that female students show more gender-awareness than their male counterparts do [39]. Risberg et al. found that key male members of faculties of medicine find sex and gender-related issues important, but of low status and do not consider them as a very relevant medical competence, which is in line with our findings [40], [41]. 

There are no significant differences in the ratings of students with and without children, migration background, disabilities or different age groups.

Again, this is an observation which is independent of the kind of undergraduate medical program in which they are enrolled. Furthermore, the students´ evaluations demonstrated that the sex/gender-sensitive and culture-sensitive medical competencies are to a large degree implemented into the new competency-based undergraduate medical program. This contrasts to their low rating in the previous traditional medical curriculum. The ratings here do not differ between female and male medical students. This is well in line with the students’ ratings on the degree of feeling prepared for the workplace, which can serve as an overarching, integrative measure for the competencies conveyed by an undergraduate medical study program. The improved and better implementation of sex/gender and culture-sensitive competencies together with the gains in general medical competencies, resulted in a better rating of the preparedness for the final clerkship year and the work as physician by students from the new, competency-based curriculum. 

In a previous study, we have shown that sex and gender competencies are structurally well implemented into the New Modular Curriculum of Medicine [31]. The high ratings of the implementation by the students clearly adds to the conclusion that this intervention was successful and that the applied approaches of a gender and diversity change agent, a ten-step approach and an institutional framework for the integration were indeed effective. In addition, one should not conclude from this study that the integration of sex/gender and cultural-sensitive competencies is dependent from the context of a competency-based undergraduate medical program. We think that with a similar systematic approach this would also be possible in a traditional, discipline-based medical curriculum. It is rather the case that the integration of gender and cultural-sensitive competencies becomes facilitated in the context of an integrated, competency-based curriculum. 

For future developments, one should bear in mind that the field of diversity-related competencies is still evolving [42], [43]. In Europe for instance, the process has been supported by a position paper published by the Standing Committee of European Doctors emphasizing the importance of the integration of gender and sex aspects into medical education and the impact on the quality of medical care [http://www.cpme.eu/cpme-policy-on-sex-and-gender-in-medicine/]. Furthermore, 207 gender and diversity learning objectives were developed and proposed by the Committee on Gender and Diversity in Medical Education of the German Association for Medical Education for the National Competence Based Catalogue of Learning Objectives for Undergraduate Medical Education (NKLM). Eighty two gender and diversity learning objectives were integrated, a yield representing 4% of all learning objectives. This catalogue aims to provide guidance for the curricular integration for medical faculties in Germany [http://www.nklm.de]. There is also an International Community of Practice "Diversity, Equity and Inclusion in Medical Education" within the AMEE (International Association for Medical Education) that tries to enhance the further integration of those issues [https://amee.org/home]. A study in the Netherlands on the inclusion of diversity into a medical curriculum has demonstrated that, although there was a supportive climate, the diversity-responsiveness of teaching material is yet to be improved [44].

Moreover, there are several efforts underway to better integrate diversity, especially sex and gender aspects, into university didactics, using sex/gender and diversity-sensitive teaching material, sex/gender and diversity sensitive language and taking differences in learning progress and behaviour of women and men as well as other diverse social groups into account [45], [46]. They also need to be integrated into study program structures providing a diversity-sensitive study environment such as family-friendly study schedules and barrier-free access to teaching facilities as well as sex/gender and diversity-sensitive admission processes. As Verdonk and Janczukowicz describe, it is necessary to “fix the content, the institution and the numbers” [43]. Different quality management and assurance instruments can be developed and used in order to support the integration and the sustainability of integrated diversity, especially sex and gender aspects like e.g. accreditation processes, student evaluations and graduate surveys [47], [48]. Along with that, items on sexual orientation, family care tasks, migration background, disabilities, family status and economic status should be integrated into student evaluations and graduate surveys to be able to analyse the needs of diverse student groups [47]. In addition, with the recent act on the integration of a further sex/gender option (“diverse”) assigned at birth by the German Federal Constitutional Court, there is a growing demand for knowledge and research on the health of Lesbian, Gay, Bisexual, Transgender, and Intersex Populations that also needs to be integrated into medical curricula [49], [http://www.bverfg.de/e/rs20171010_1bvr201916.html]. Data on the health of this population group are at present limited and efforts are being made to integrate this dimension into national health surveys in order to collect epidemiological data [50].

This study also has limitations that should be transparent to the reader. First, it is a single centre study that needs additional research to show generalizability of the findings in regard to other institutions and contexts. Secondly, although a substantial absolute number of students did participate in the study, the relative response rate was only 22%. This may involve a bias in the selection of the students with a potential effect on the study results. Our response-rate should be seen in the light that it is in the range usually achieved with email-initiated surveys [51]. Thirdly, we do not have any evidence in regard to which extent the integrated sex/gender- and culture-sensitive competencies improve the ratings of the preparedness. Finally, the positive ratings of the curricular integration of sex/gender and culture-sensitive competencies into the new curriculum does not allow the conclusion that students from the new curriculum actually apply those competencies in their future workplace.

## 5. Conclusions

The present study provides empirical evidence that medical students in their final clerkship year rate sex/gender and culture-sensitive competencies as highly relevant for their future work as a physician. Furthermore, their ratings provide additional and complementary indications that a successful integration of sex/gender and culture-sensitive competencies can in fact be achieved by a systematic structured approach applied for the implementation. Overall, this study demonstrates the need to implement diversity-related competencies into undergraduate medical education in general. Given that issues of diversity are continuously evolving in research and practice, this implementation should allow dynamic adaptations to be made in line with the current state-of-the-art. 

## Acknowledgements

We would like to thank the Vice Dean of Student Affairs Prof. Dr. Adelheid Kuhlmey and the Dean of Student Affairs Prof. Dr. Joachim Spranger, the Director of the Teaching and Learning Division Burkhard Danz, Prof. Dr. Markus Feufel, former advisor to the Vice Dean of Student Affairs as well as our colleagues from the quality assurance section Martina Klau-Fadke, Rita Kraft, Peter Kube, Mandy Petzold, Dr. Yadira Roa-Romero and from the Dieter Scheffner Center Josefin Bosch, Andreas Böttner, Dr. Anja Czeskleba and Ylva Holzhausen for their continuous support and contribution to the study.

## Competing interests

The authors declare that they have no competing interests. 

## Figures and Tables

**Table 1:**
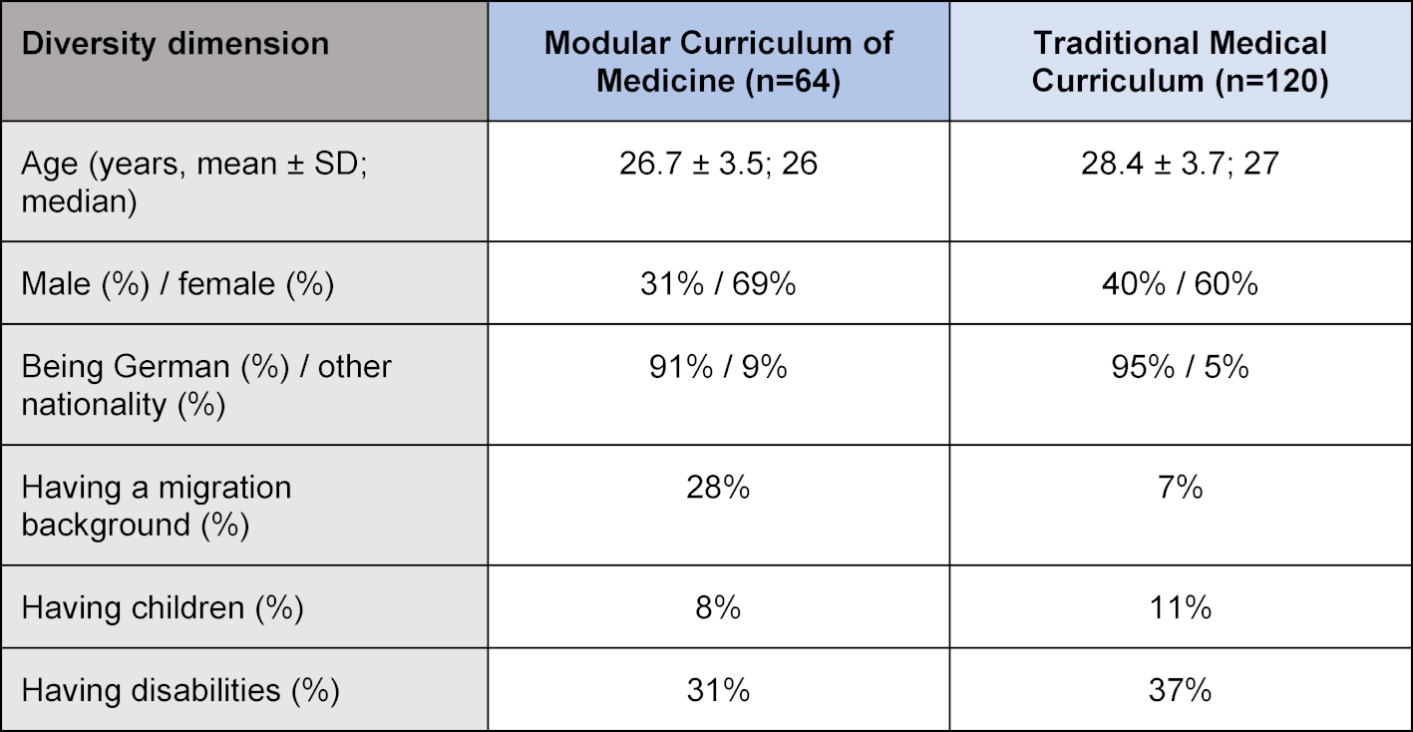
General demographic information
